# Supplementary figures and images for: Development and Application of a Cleaved Amplified Polymorphic Sequence Marker (Phyto) Linked to the Pc5.1 Locus Conferring Resistance to Phytophthora capsici in Pepper (Capsicum annuum L.)
Source: Plants (Basel). 2023 Jul 25;12(15):2757. doi: 10.3390/plants12152757 (PMC10421461; doi:10.3390/plants12152757)

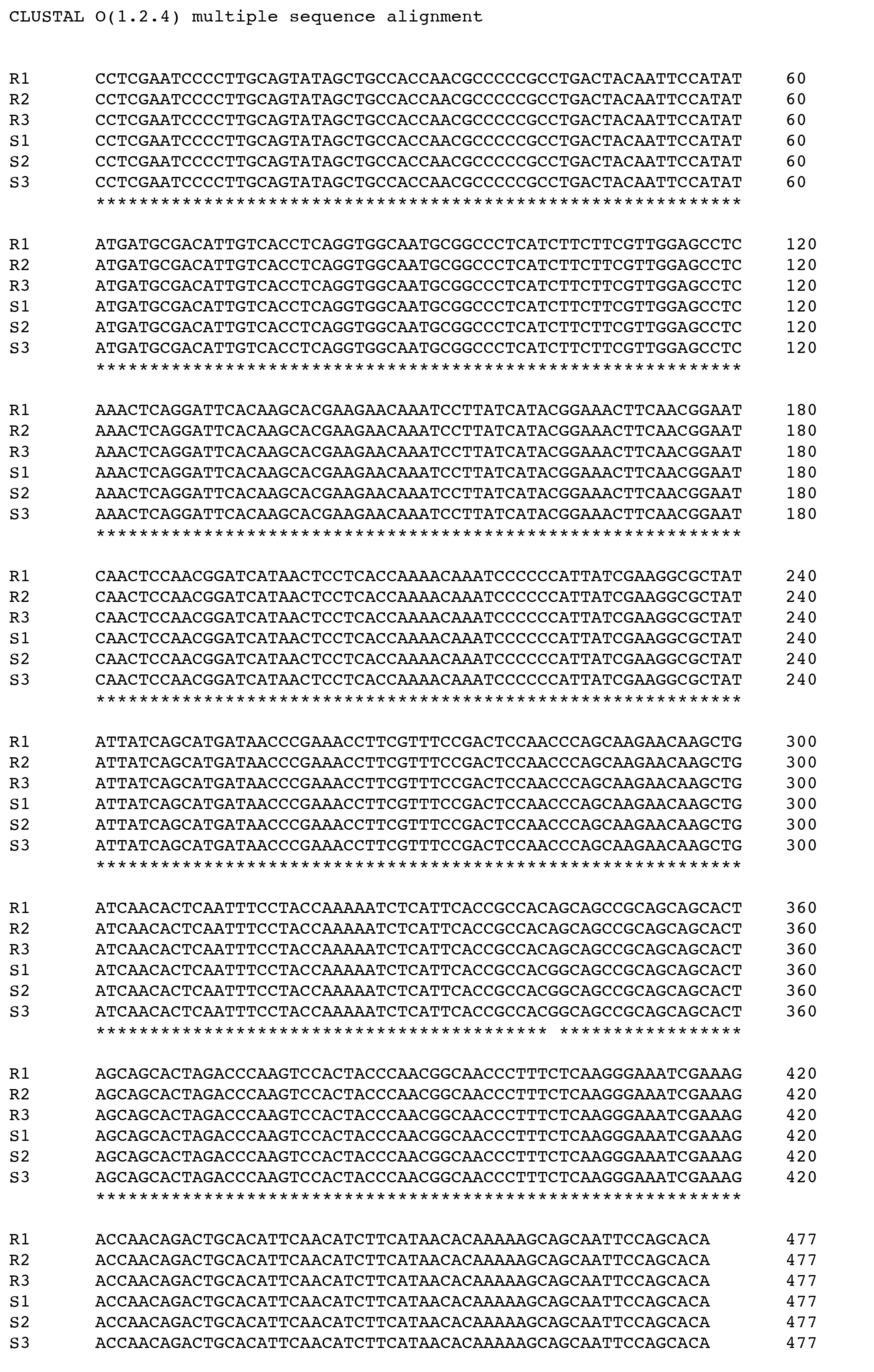

Supplement: Supplementary file 1 [file plants-12-02757-s001.zip › supplementary figure S1.tif]
